# Experimental and Theoretical Studies on Possibility of Void Filling by Magnesium in Mg-Doped Tetrahedrites

**DOI:** 10.3390/ma15124115

**Published:** 2022-06-09

**Authors:** Juliusz Leszczyński, Krzysztof Kapera, Adrian Mizera, Paweł Nieroda, Andrzej Koleżyński

**Affiliations:** 1 Department of Inorganic Chemistry, Faculty of Materials Science and Ceramics, AGH University of Science and Technology, al. A. Mickiewicza 30, 30-059 Krakow, Poland; amizera@agh.edu.pl (A.M.); pnieroda@agh.edu.pl (P.N.); 2 Department of Silicate Chemistry and Macromolecular Compounds, Faculty of Materials Science and Ceramics, AGH University of Science and Technology, al. A. Mickiewicza 30, 30-059 Krakow, Poland; kaperak@agh.edu.pl (K.K.); andrzej.kolezynski@agh.edu.pl (A.K.)

**Keywords:** thermoelectrics, tetrahedrites, SPS sintering, void filling, electronic structure calculations

## Abstract

Tetrahedrites, due to their promising thermoelectric properties, are one of the materials being investigated for use in thermoelectric generators. One problem is the lack of *n*-type tetrahedrites, which would be beneficial for the design of tetrahedrite thermoelectric modules. Preliminary theoretical studies have shown that elements from groups I and II can be introduced into the structural voids of tetrahedrite, acting as donor dopants, and should enable *n*-type conductivity. Therefore, in this work, an attempt was made to obtain and study magnesium-doped tetrahedrites. A series of samples, Mg_x_Cu_12_Sb_4_S_13_, with different magnesium contents were obtained and their phase and chemical compositions were characterized. It was observed that the structural changes occurring upon doping indicate that Mg atoms are likely to be embedded in the structural voids. The experimental studies have been supported by electronic structure calculations indicating that the most likely location of Mg is in the structural voids at the 6b Wyckoff position. Seebeck coefficient and resistivity measurements showed that doping with Mg reduces the concentration of holes, which is consistent with the predicted donor character of the dopant. However, the introduction of magnesium in sufficient amounts to achieve *n*-type conductivity was not successful.

## 1. Introduction

**Figure 1:**
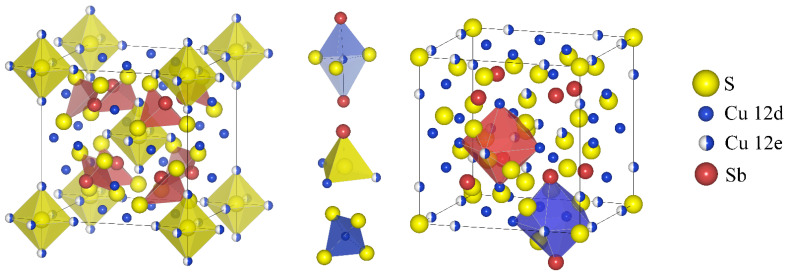
Crystal structure of tetrahedrites with main polyhedra building blocks.

In previous years, the interest of several leading research groups has focused on materials in the tetrahedrite group. They occur naturally as a mineral with the formula Cu_12_Sb_4_S_13_. They crystallize in the cubic crystal system, space group I-43m, and have a complex crystal structure with a unit cell of 58 atoms, presented in Figure 1. The source of the very low thermal conductivity of tetrahedrites (~0.5 W·m^−1^·K^−1^ T > 300 K) is anharmonic vibrations of Cu atoms with trigonal coordination due to interactions with the lone pair of electrons located at the antimony atom. The low thermal conductivity is a very desirable feature in high performance thermoelectric materials for energy conversion. It is, among other things, the low thermal conductivity that has led to great interest in tetrahedrites and intensive research on these compounds. The undoped tetrahedrite Cu_12_Sb_4_S_13_ is a *p*-type semiconductor showing metallic character with high electrical conductivity of 10^5^ S·m^−1^ at 300 K and a moderately high Seebeck coefficient, which together with very low thermal conductivity values allows for achieving a *ZT* parameter of 0.56 (at T = 673 K) [1]. Appropriate doping allows an increase in *ZT_max_* of tetrahedrites, which has been shown in many works [2,3,4,5,6]. Due to the high carrier i.e., electron holes, concentration of pure tetrahedrite, mainly dopants and structural modifications reducing the hole concentration, i.e., donor-type dopants, are used to increase the *ZT* value. The second group of modifications includes isovalent dopants, with the main purpose of decreasing thermal conductivity. Among the modifications of the Cu_12_Sb_4_S_13_ structure described in the literature, we can find: doping of s-, d-, *p*- block elements in the Cu sublattice (Cu_12−x_T_x_Sb_4_S_13_, where T = Mg, Fe, Cd, Co, Mn, Zn, Ni, Ge, Sn) [1,2,3,4,5,7,8,9,10,11]; *p* block elements doping in Sb sublattice (Cu_12_Sb_4−x_M_x_S_13_, M = Bi, Te [12,13,14]); selenium doping in sulfur sublattice (Cu_12_Sb_4_S_13−x_Se_x_ [15]); sulfur deficiency (Cu_12_Sb_4_S_13−x_ [15]); and excess Cu (Cu_12+x_Sb_4_S_13_ [16]) in structural voids at Wyckoff position 24g. An additional result of the aliovalent doping may be a reduction in the thermal conductivity of pure tetrahedrite from about 1 W·m^−1^·K^−1^ to about 0.4 W·m^−1^·K^−1^ [10,17]. As a result, the highest reported *ZT* values for doped *p*-type tetrahedrites exceed 1 (*ZT*_max_ = 1.13 at T = 575 K for Cu_11_MnSb_4_S_13_ [11]) and indicate that they can be a viable alternative to lead-containing acceptor type materials based on PbTe in the temperature range of 570–670 K [18]. Although many dopants with a donor character have been studied, so far it has not been possible to find one that allows a change in electrical conductivity to the *n*-type. Obtaining *n*-type tetrahedrites with good thermoelectric properties would be beneficial for constructing thermoelectric modules based on the tetrahedrites. The use of *p*- and *n*-type tetrahedrites in such modules would eliminate many design problems resulting from differences in thermal expansion coefficients and the selection of different materials for diffusion barriers and metallic contacts.

The substitution of native atoms by dopant atoms leads, in addition to transport changes, to noticeable variations in the crystal structure, the most obvious of which is a change in the lattice parameter. The change in this parameter resulting from changes in interatomic distances is also one of the pieces of evidence of dopant incorporation into the structure. For d-block metal dopants, it was experimentally proved that they substitute Cu^2+^ cations at the 12d position. This is in agreement with theoretical findings, which predicts preference of the 12d Wyckoff position for multivalent dopants, while for the monovalent dopants e.g., Ag, the 12e site of trigonally coordinated Cu would be preferred. For most d-block dopants introduced in the copper position, the lattice parameter increases. This is especially so for the Cd dopant, which for a compound with the formula Cu_12−x_Tr_x_Sb_4_S_13_ for x = 1.5, results in an increase in the lattice parameter *a* to 10.47 Å (1.047 nm) [3]. A similar increase in the lattice parameter can be observed for the Mn [19] dopant, where for x = 1.8, the lattice parameter is higher than 10.43 Å. An increase in the lattice parameter to about *a* = 1.038 nm for x = 2 can be observed for Zn doping. For Co doping, the lattice parameter increases slightly to about *a* = 10.34 Å [4], while for Ni doping the lattice parameter hardly changes, 10.319–10.323 Å [2]. These changes are consistent with the ionic radii of the listed divalent atoms decreasing in the series Cd, Mn, Zn, Co, Ni, and if we assume a 2+ charge of the ion, they are all larger than the Cu^2+^ ionic radius. Similar results were observed for elements other than d-block metals in the copper site, such as *p*-block elements Sn (*a* = 10.375 Å) and Ge (*a* = 10.339 Å) [19]. The open structure of tetrahedrite also allows atoms to be inserted into structural voids [16,20]. Vaqueiro et al. investigated the effect of excess copper introduced into structural voids for Cu_12+x_Sb_4_S_13_ (0 ≤ *x* ≤ 2) and showed that it is possible [16]. The introduced excess copper occupies void 24g site (0.28, 0.28, 0.041) and above 393 K becomes mobile, with the whole material showing superionic conductivity. Similar atomic coordinates for excess copper (0.29, 0.29, 0.03) were determined in single crystal studies by Makovicky and Skinner [20]. As expected, the introduced excess copper decreased electrical conductivity and increased the Seebeck coefficient of all samples compared to the undoped material, with simultaneous decrease of thermal conductivity, which lead to the maximum value of *ZT*_max_~0.6 (*T* = 573 K) for Cu_14_Sb_4_S_13_ [16]. The authors suggest that the exceptionally low thermal conductivity for excessive copper tetrahedrites is consistent with the assumptions of the PLEC theory (phonon liquid electron crystal) valid for the superionic materials. Changes in the lattice constant, as can be seen, are an important parameter in doping studies, and the results presented here provide an important reference point in our research.

All of these results clearly indicate that the introduction of dopants can be an effective way to optimize the transport properties of tetrahedrites and thus *ZT* parameter increase. These results also show that it is possible to introduce into the structural voids an excess of atoms/cations with small ionic radii, such as Cu. This possibility inspired us to search for a dopant that could be introduced as a small cation into structural voids. As potential dopants we considered elements of groups I and II of the periodic table, due to their high susceptibility to ionization and small ionic radii. Both simple considerations based on electron count and first rough DFT calculations performed by us showed that such introduction of atoms into the voids will lead to the formation of donor states, which, at appropriate concentration, may lead to the formation of *n*-type material. In the case of divalent metals such as Mg or Ca, this should occur at two times lower dopant concentration compared to monovalent metals such as Li or Na. Moreover, in the meantime, a paper devoted to Mg-doped tetrahedrite Mg_x_Cu_12−x_Sb_4_S_13_ was published by Levinski et al. [7] and a surprisingly large increase in the lattice parameter upon doping was observed (*a* = 10.40 Å for x = 1.5). In our opinion, this increase is difficult to explain by Mg substitution for Cu, due to the fact that the ionic radius of Mg is equal or slightly smaller than that of copper, and suggests that this lattice parameter increase may be the result of the filling of structural voids in the tetrahedrite structure. Although, in the aforementioned work, it was assumed that Mg substitutes Cu, and the change in lattice parameter was attributed to the incorporation of Mg into the tetrahedrite structure, the exact localization of Mg in the structure was not experimentally determined. Therefore, we decided to check if the novel concept of introducing a dopant into the structural voids of tetrahedrites is possible and if in this way it will be possible to obtain an *n*-type conductivity tetrahedrite, which has not previously been obtained. We have selected Mg as the dopant for the reasons outlined above and due to the tendency of magnesium to form non-directional bonds with spherical symmetry, which may additionally result in a decrease in thermal conductivity, thereby increasing *ZT*. A series of Mg_x_Cu_12_Sb_4_S_13_ tetrahedrite samples were synthesized and compacted and selected thermoelectric parameters were investigated. These studies were combined with ab initio theoretical calculations of the electronic structure and electron density topology.

## 2. Materials and Methods

### 2.1. Ab Initio Calculations

Ab initio calculations of electronic structure and electron density topology were performed with the WIEN2k computational package for a series of model structures. WIEN2k employs Full Potential Linearized Augmented Plane Wave (FP-LAPW) approximation within Density Functional Theory formalism, which is well suited to solids. For all WIEN2k calculations, the following parameters were used: GGA PBEsol as exchange-correlation potential, cutoff parameters R_MT_K_MAX_ = 8 and G_MAX_ = 20, 256 k-points in irreducible Brillouin zone for unmodified structure (and closely matching number of k-points for other structures) and convergence criteria of ΔE = 10^−5^ Ry for energy, Δq = 10^−5^ e for charge and ΔF = 10^−1^ mRy a_0_^−1^ for forces. These data were further processed using Brown’s Bond Valence Model (BVM) for a more global view of the structure’s properties, including strain factor and global instability index.

### 2.2. Synthesis

Stoichiometric amounts of the substrates were weighed and inserted into quartz ampoules in a glove box under anaerobic conditions. In the course of the research, it was found that the best results are obtained by conducting a multi-step synthesis by direct solid-phase reaction with a preliminary melting step. The ampoule was placed in a rocking furnace for 24 h at 940 K. Due to the decomposition of the tetrahedrite phase to Cu_3_SbS_4_ and Cu_3_SbS_3_ at temperatures above 720 K, the furnace was cooled to 720 K and the ampoules were annealed at this temperature for one week. The resulting ingots were crushed, resealed in quartz ampoules and annealed for two weeks at 720 K.

### 2.3. Structural, Microstructural and Chemical Characterization

The phase composition was determined based on X-Ray Diffraction (XRD) measurements using Phillips X’Pert Pro diffractometer (CuK_α1_ = 15,406 Å, 2θ = 10–120°). The Rietveld refinement implemented in HighScore software was used to determine lattice parameters, atomic positions and phase weight fractions. Because of the average goodness of fit (GoF) (GoF ≈ 1.9 for as synthesized and GoF ≈ 8.3 for sintered samples) other parameters were not considered. The microstructure of produced materials was analyzed using two scanning electron microscopes (SEM) coupled with an energy-dispersive spectrometer (EDS), which allowed analysis of the chemical composition of selected points/areas of the sample. Qualitatively, the EDS analysis results obtained from both instruments were in agreement with each other, but in this paper we present the results obtained only from the Thermo Fisher Scientific Phenom XL scanning electron microscope due to smaller errors in the quantitative analysis of the chemical composition. During the analysis, it was found that accurate quantification of the chemical composition, in both point and surface analysis, is only possible for areas having precipitates of impurity phases, such as Cu_2_S and MgS. In other cases, systematic errors did not allow for obtaining reproducible and quantitatively consistent results of composition analysis. For this reason, the specimen names we use in this paper correspond to their nominal compositions.

The established procedure made it possible to obtain practically pure Cu_12_Sb_4_S_13_ and Mg-doped Mg_x_Cu_12_Sb_4_S_13_ with small amounts of impurities, which was confirmed by XRD studies and SEM observation with EDS composition analysis.

### 2.4. Sintering

Subsequently, the selection of densification conditions of the obtained powders by Field Assisted Sintering/Spark Plasma Sintering (FAST/SPS) was investigated. They showed that there are no single universal densification parameters for pure and doped tetrahedrite. When using higher temperatures >430 °C and pressures of the order of 50 MPa, the densification time was about 2 min, but severe cracking of the samples was observed after the process. When the temperature was lowered to below 380 °C, densification of the material below 80% of the theoretical density combined with low mechanical strength was observed. For pure tetrahedrite, the optimal sintering conditions were found to be 430 °C and 50 MPa for 15 min, yielding densities above 95% of the theoretical density. In Mg-doped tetrahedrites, the conditions were set at: T = 400–420 °C, depending on the dopant concentration; *p* = 30 MPa, t = 15–20 min, depending on the observed sintering curve.

### 2.5. Electrical Characterisation

The Seebeck coefficient was measured using steady state conditions and variable temperature gradient across the specimen up to 5 K. Electrical resistivity was measured using a four-probe method using variable DC polarization. Both of these measurements were carried out in the same apparatus during heating and cooling cycle on cylindrical specimens 10 mm in diameter and 12 mm long. Measurements were carried out in the temperature range from the room temperature to 680 K. Results shown are for both heating and cooling cycle. Experimental error was below 5% for the Seebeck coefficient measurements and 1.5% for the electrical resistivity measurements.

## 3. Results and Discussion

### 3.1. Theoretical Calculations

**Figure 2:**
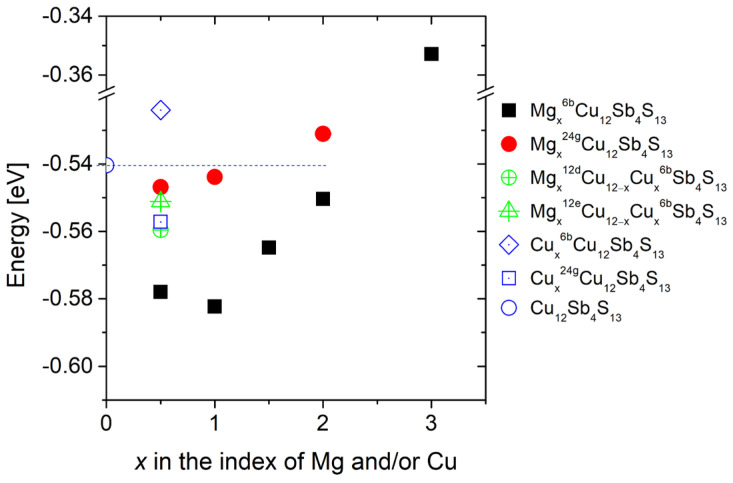
Calculated energy of formation of Mg_x_Cu_12_Sb_4_S_13_ and Cu_12+x_Sb_4_S_13_. Dashed line indicates energy for pure tetrahedrite. The upper indexes indicate the position of the atom in the structure for which calculations were carried out. If not indicated, Cu, Sb and S occupy their nominal positions, i.e., 12d/12e, 8c, 24g/2a, respectively.

A series of doped tetrahedrite structures, Mg_x_Cu_12_Sb_4_S_13_, for x = 0.0, 0.5, 1.0, 1.5, 2.0 and 3.0. was created. In those structures, magnesium atoms were introduced into two different structural voids, described by Wyckoff’s 6b ($0\;\frac{1}{2}\;\frac{1}{2}$) and 24g (x x z) positions. In addition, structures were created in which dopant Mg atoms substituted Cu atoms at positions 12e or 12d, which, in turn, occupied structural voids at the 6b site, and two structures of composition Cu_12+0_._5_Sb_4_S_13_ with excess Cu atoms at positions 6b and 24g. In total, 12 different structures were analyzed. DFT calculations (by the means of WIEN2k, as mentioned in Section 2.1) for pristine and for doped structures led to the optimization of lattice parameters and the atomic positions of these structures with respect to forces, in a way that minimized total energy of the structure. Using the total energy of fully optimized and relaxed structures, energy of formation *E_Form_* was calculated, which is defined as the energy difference between the total energy of a given structure and the sum of total energies of pure element structures, in their thermodynamically stable forms. For instance, for Mg_x_Cu_12_Sb_4_S_13_:

(1) $$E_{Form}^{{\mathrm{Mg}}_{x}{\mathrm{Cu}}_{12}{\mathrm{Sb}}_{4}S_{13}}=E_{tot}^{{\mathrm{Mg}}_{x}{\mathrm{Cu}}_{12}{\mathrm{Sb}}_{4}S_{13}}-12\cdot E_{tot}^{\mathrm{Cu}}-4\cdot E_{tot}^{\mathrm{Sb}}-13\cdot E_{tot}^{S}-x\cdot E_{tot}^{\mathrm{Mg}}$$

where both *E_Form_* and *E_tot_* are in eV/atom units. The results of the calculations of the energy of formation show (Figure 2) that the most energetically favorable structures are those in which magnesium is located in structural voids in Wyckoff’s position 6b in amounts of 0.5 and 1 atoms per unit cell. The introduction of larger amounts of magnesium significantly raises the formation energy. This may suggest that the solubility limit of magnesium in the tetrahedrite structure will exist for a concentration of Mg less than sufficient to fill all structural voids in the 6b position. Further, energetically favorable are structures in which magnesium replaces copper at positions 12d and 12e and, simultaneously, copper occupies position 6b. A similar energy of formation has a system in which one copper atom per unit cell occupies the □^24g^ void. In the case of copper inserted in the 6b or 24g voids, it is clear that the latter is energetically more favorable. This result is in agreement with experimental results from the work of Vaqueiro et al. [16] and Makowitzki et al. [20], confirming the correctness of the performed calculations. On the other hand, comparing the formation energy of structures with Mg in the 6b voids with *E_Form_* of structures with Cu in the 24g voids, it can be seen that the structures with Mg are energetically more favorable.

**Figure 3:**
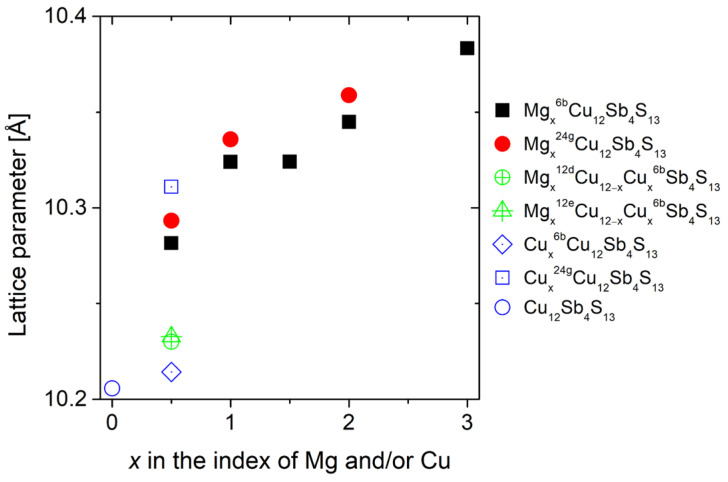
Calculated lattice parameters of Mg_x_Cu_12_Sb_4_S_13_ and Cu_12+x_Sb_4_S_13_. The upper indexes indicate the position of the atom in the structure for which calculations were carried out. If not indicated, Cu, Sb and S occupy their nominal positions, i.e., 12d/12e, 8c, 24g/2a, respectively.

Introducing atoms into the voids also changes the lattice parameter (Figure 3). In the case of the introduction of 0.5 magnesium atoms per unit cell in the 6b void, there is a relative increase in the lattice parameter of about 0.74%, which is smaller than the increase in the lattice parameter (0.85%) in the case of the introduction of the same amount of Mg atoms in the 24g void. The lower lattice deformation is clearly combined here with a lower formation energy. The Mg atom’s preference for position 6b may be due to its greater spherical symmetry (Mg forms ionic bonds) and greater distance from the atoms forming the coordination polyhedron, which gives greater stability. In the case of Cu atoms introduced into the voids, the effect on the lattice parameter is similar and the increase of the lattice parameter for the dopant introduced into the void at the 24g site is about 20% higher than for the 6b site. The presence of directional *p*- and *d*-type orbitals in the bonds formed by Cu atoms with their neighbours means that the much more asymmetric environment in the □^24g^ gives greater atomic position stability.

**Figure 4:**
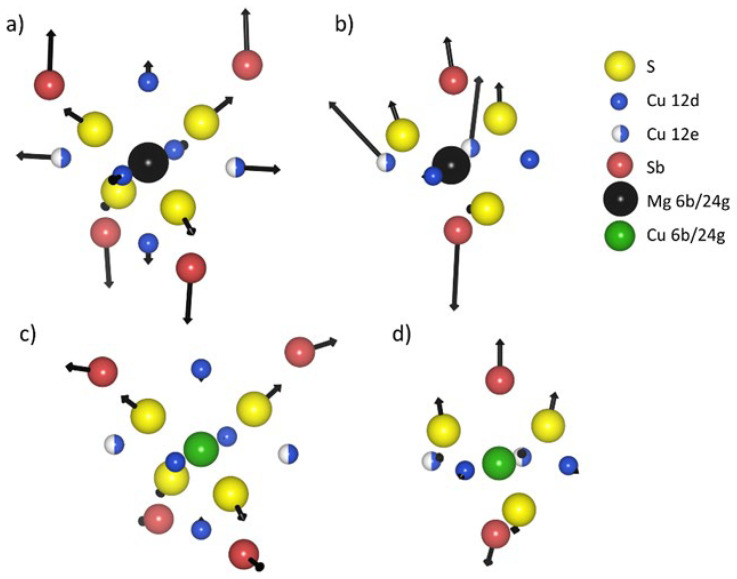
Change of atomic positions of nearest neighbors of a structural void after introducing Mg or Cu atom (Mg and Cu shown as black balls) into the void for: (**a**) Mg in 6b void, (**b**) Mg in 24g void (x = 1.0 magnesium atom per unit cell for both shown examples), (**c**) Cu in 6b void, (**d**) Cu at 24g void (x = 0.5 Cu atom per unit cell for both shown examples). The arrows indicate the direction and magnitude of the shift of the atoms relative to the primary position.

The introduction of Mg and Cu atoms into structural voids causes significant changes in the bond lengths formed by the dopant atoms, with the atoms forming coordination polyhedra. General trends in change of atomic positions can be seen in Figure 4. The magnitude of those changes slightly varies, depending on the concentration of introduced magnesium. Introducing additional copper atoms into 6b or 24g structural voids also differs in its effect. All the atoms move away from magnesium after its introduction into the structural void at the 6b Wyckoff position (Figure 4a). Cu^12d^ atoms change their position more than Cu^12e^ atoms, since their initial distance to the Mg atom was also shorter (2.54 and 2.85 Å for Cu^12d^-Mg and Cu^12e^-Mg, respectively, for the unmodified structure). The change in position of S^24g^ atoms also results in the nearest Sb^8b^ atoms having to accommodate it, causing a noticeable shift. It should be noted that due to symmetry, until x = 2 magnesium atoms are introduced, only four antimony atoms are affected, while the other four mostly keep their original coordinates. Magnesium tends to form ionic, closed-shell bonds, which fits well into the highly symmetrical 6b structural void. On the other hand, introducing copper into 6b voids would result in more directional, covalent bonds, which may be questionable given the number of other copper atoms (Cu^12e^ and Cu^12d^) in close proximity (Figure 4c). The results of enthalpy of formation clearly showed that this variant for Cu is not energetically favorable.

The structural void at Wyckoff position 6b is larger and more symmetrical than the one at the 24g position, which means that filling structural void □^24g^ with Mg should yield comparably greater changes to its local environment. This is noticeable in Figure 4b for Cu^12e^ and Sb^8c^ atoms, where the introduction of magnesium causes a large shift in their positions. It is worth noting that most atoms do not move away from magnesium in a straight line, as their local surroundings do not always allow it. This means that the introduction of magnesium in void □^24g^ possibly results in a higher order of bond strain and a structural “mismatch”. Inserting a copper atom into the □^24g^ structural void has quite a different outcome (Figure 4d). From its neighboring atoms, only antimony and sulfur yield noticeable changes in their atomic position, while Cu^12e^ and Cu^12d^ do not shift. Copper forming directional bonds is more preferable in the smaller and asymmetrical structural void □^24g^, which is in agreement with the formation energy.

The above analyses confirmed that Wyckoff’s position 6b of the void is clearly favorable for Mg atoms; thus, we will focus on theoretical calculation results for this particular structure. Analysis of the structural data calculated for relaxed structures using Brown’s Bond Valence Model allowed obtaining several global parameters of structure and bond properties, such as mismatch of the whole structure, defined as a global instability index *D* and global bond strain index δ* (Table 1).

Both the global instability index *D* and global bond strain index δ* grow with increasing Mg content. For the *D* index, one can see a huge increase between x = 0.5 and x = 1. A similar jump can be observed for the global strain index between x = 1 and x = 1.5. The bond strain index *δ* for particular bonds (Table 1) follows the trend of the global strain index and increases with the amount of magnesium introduced. While, for x = 0.5, it is still relatively small for all bonds, for x = 1, the strain increases significantly for the Cu_1_-Sb and S_1_-Cu_3_ bonds, and for x > 1, it increases strongly for the other bonds, which explains in part the increase in formation energy for structures Mg_x_^6b^Cu_12_Sb_4_S_13_ with x > 1.

**Figure 5:**
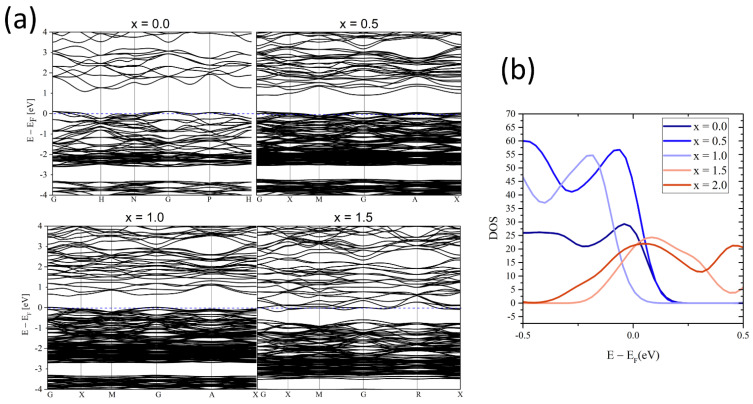
Calculated electronic band structures (**a**) and density of states (**b**) for Mg_x_Cu_12_Sb_4_S_13_.

The introduction of magnesium into the voids affects the electronic structure of the resulting material, which can be observed by analyzing the evolution of the band structure of tetrahedrites during doping (Figure 5). Since Mg doping in the interstitial sites introduces an additional two electrons for each Mg atom, hole compensation and increase of Fermi level occur, which is accompanied by a decrease in electron hole concentration for x < 1, and for x > 1 the Fermi level is shifted to the conduction band and the electron concentration increases. The introduction of Mg atoms in amounts higher than 1 at./formula unit (f.u.) should allow for obtaining *n*-type conductivity in tetrahedrites, both in the case of the introduction of Mg atoms into 6b voids and into 24g voids. Additionally, it can be observed that under doping the value of the energy gap decreases, which in the extreme case, for x = 3, can lead to semi-metallic properties (Figure S1 in Supplementary Materials). Simultaneously, with the decrease in the energy gap, significant changes in the position of the extrema in the lowest-lying conduction bands occur, resulting from the large contribution of Mg *s*-type character orbitals, Sb *p*-type character orbitals and Cu *d*-type character orbitals. Modifications of the highest bands in the valence band are much smaller than in the conduction band and concern mainly the *H* point and its surroundings.

### 3.2. XRD Results

**Figure 6:**
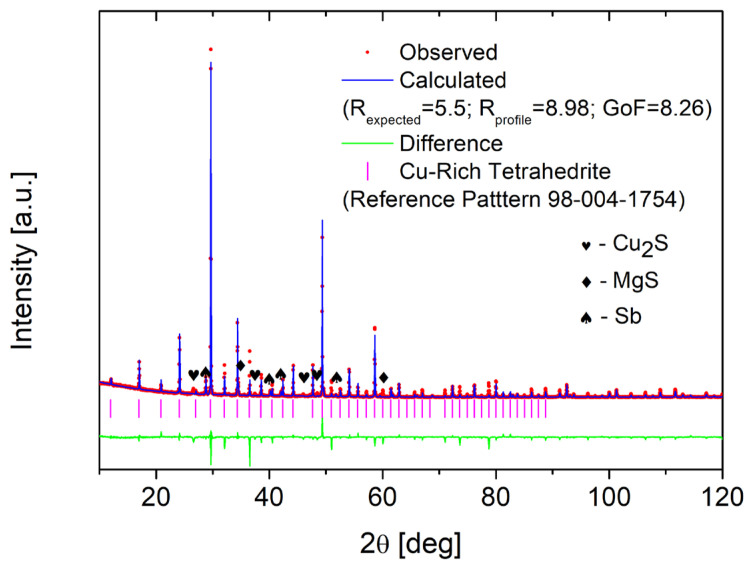
Example observed x-ray diffraction pattern with pattern calculated from Rietveld refinement for Mg_0.5_Cu_12_Sb_4_S_13_ sintered sample.

**Figure 7:**
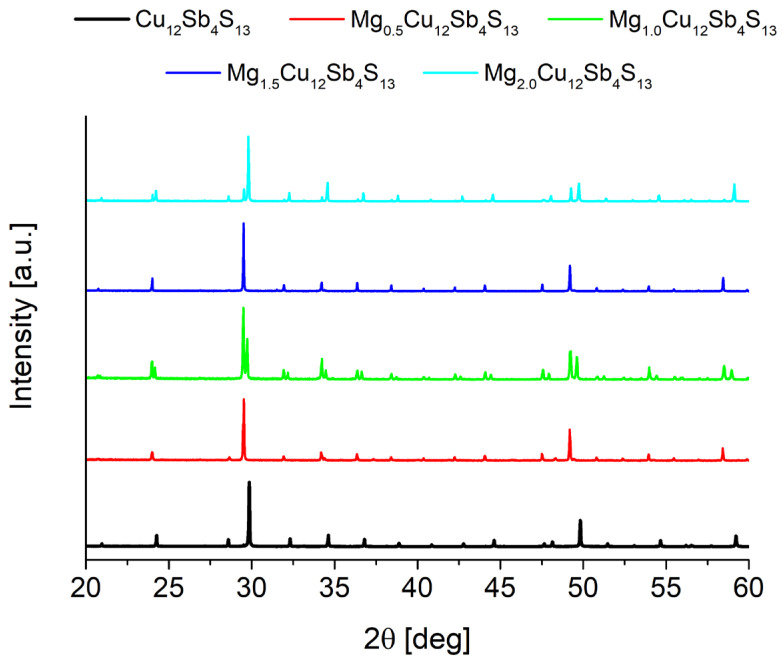
XRD diffraction patterns of Mg_x_Cu_12_Sb_4_S_13_ specimens after synthesis.

**Figure 8:**
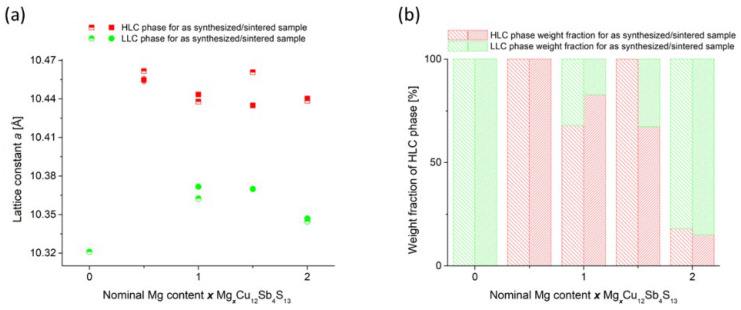
Lattice constants measured for tetrahedrite phases present in Mg_x_Cu_12_Sb_4_S_13_ samples after sintering (**a**) and weight fraction of the high lattice constant phase in these samples (**b**).

Structural XRD investigations show that all the samples obtained consist predominantly of the tetrahedrite phase. Impurities in small amounts of MgS, Sb and Cu_2_S appear (Figure 6). As can be seen in Figure 7, the doping caused a clear shift in the position of the reflections and therefore affected the change in the lattice parameter. Similar to the study of Vaqueiro et al. [16], the doping of Mg caused an increase in the lattice parameter and at the same time a decomposition into two phases with a tetrahedrite structure: the first phase with a high lattice constant (HLC) and the second phase with a lower lattice constant (LLC). The lattice constant of the HLC tetrahedrite phase is very similar (10.45 Å compared to 10.44 Å) to those in the case of the copper-rich phase partially filled with Cu at □^24g^. The increase in the lattice parameter for the HLC phase is approximately 1.25%. In contrast to Vaqueiro’s work, the lattice parameter of this second phase is larger than that of pure tetrahedrite and is about 10.37 Å, indicating some dopant incorporation into this tetrahedrite structure. These results also differ from the observations in the work of Levinski et al. [7], who observed a linear increase in the lattice parameter in Mg_x_Cu_12−x_Sb_4_S_13_. The content of the HLC phase is the highest for the sample with nominal composition x = 0.5 (Figure 8). Increasing the Mg content leads to a progressively higher proportion of the LLC phase. The XRD results for the post-synthesis powders as well as for the sintered specimens are very similar, both in terms of lattice parameter, atomic positions, and the phase content. This shows that the presence of two phases is not likely due to the high temperature phase quenching and blocking of the phase decomposition/transformation. The results of XRD measurements for both as synthesized and sintered specimens were subjected to Rietveld analysis in order to accurately determine the structural changes and explain their source.

Unfortunately, simulations of XRD patterns show that the insertion of Mg into the copper position as well as into the voids □^24g^ and □^6b^ induces non-specific changes in reflection intensity, which does not allow us to unambiguously determine the position into which Mg is incorporated. It also does not allow us to determine whether it is not by chance that the Cu atom occupies the □^24g^ or □^6b^ voids. Different structural models were used in the Rietveld refinement: Mg at □^6b^, Mg at □^24b^, Cu at □^6b^, Cu at □^24g^ site, substitution of Cu by Mg at both sites Mg_Cu_^12e^, Mg_Cu_^12d^, etc. A characteristic feature common to all these models was that during refinement, the site occupancy of atoms filling structural voids decreased to zero. The fitting parameters were not very high, and the calculations often became divergent, usually leading to physically meaningless ADP parameter values. Similar to the work of Vaqueiro et al. [16], in the case of a HLC phase, the occupancy of Cu sites was clearly lower than 1. This may indicate a large disorder in the copper sublattice, as in the case of a tetrahedrite partially filled with copper, or a partial copper substitution with magnesium atoms.

**Figure 9:**
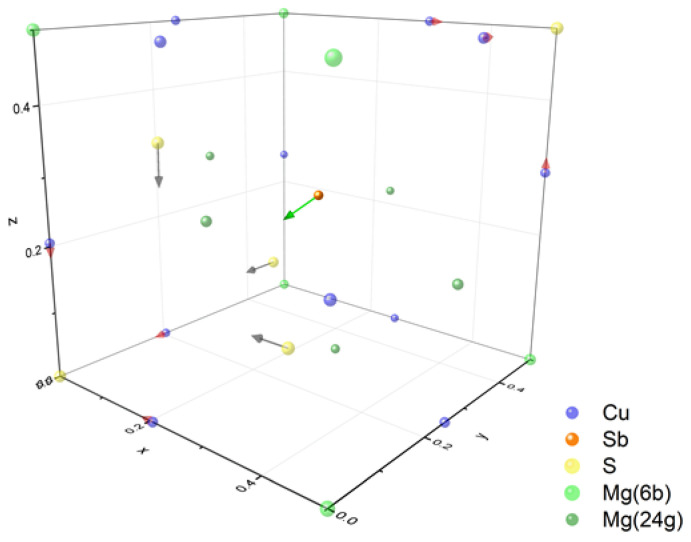
Example plot of atomic position shifts (arrows show 10× multiplied atomic position shift) for Mg_0.5_Cu_12_Sb_4_S_13_ sample (pure Cu_12_Sb_4_S_13_ sample is used as a reference).

**Figure 10:**
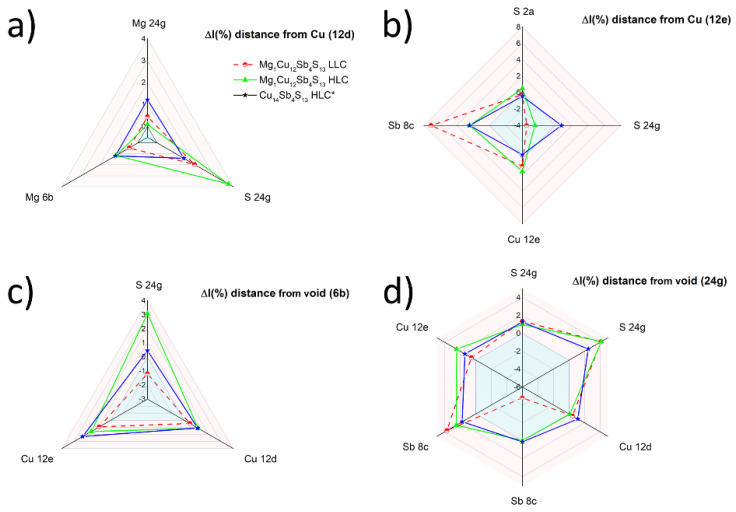
Plots of the relative change in bond length or distance Δl upon doping with Mg meas-ured at different atomic sites for Mg_1_Cu_12_Sb_4_S_13_. (**a**) Cu 12d site, (**b**) Cu 12e site, (**c**) void 6b site, (**d**) void 24g site. Results are expressed as a percentage of a given bond/distance. * Results for Cu_14_Sb_4_S_13_ from [16].

Indirect evidence for the location of the dopant can be provided by a shift in the position of the atoms in the unit cell referred to the pure tetrahedrite phase. Figure 9 shows the shift in the average position of the atoms with respect to the pure tetrahedrite measured for the HLC phase in Mg_0_._5_Cu_12_Sb_4_S_13_. We can observe that the largest shifts are experienced by the S^24g^ atoms, which move towards position 2a, i.e., the corners of the unit cell and its center, and by the antimony atoms, half of which move towards the center of the unit cell and half towards the corners of the unit cell. Because of the increasing lattice constant, the distances between S^2a^ and Cu^12e^ also increase. If we look at the position of the structural voids □^6b^ and □^24g^, we can see in both cases, the distances to adjacent atoms increase. A similar picture can be observed for the other samples for the HLC tetrahedrite phase. These shifts can also be presented as a change in average bond length around selected atomic positions. Because Mg shows the greatest similarity in chemical properties and ionic radius to Cu, among the elements that form tetrahedrite, it can be assumed that the Mg atoms will either take the place of the copper atoms in the tetrahedrite structure or, as follows from theoretical calculations, will occupy the structural voids □^6b^, or less likely, □^24g^. The relative distance/bond length change for selected atomic sites in Mg_1_Cu_12_Sb_4_S_13_ is presented in Figure 10. In these plots, we have included results calculated for the Cu_14_Sb_4_S_13_ structure refined by Vaqueiro et al. [16]. The results for the remaining samples are included in Supplementary Materials (Figure S2).

In the case of the Cu^12d^ site (Figure 10a), the Cu^12d^-S^24g^ bond lengths increase, and the increase is larger for HLC phases and amounts to about 4%. Significantly lower bond elongation with S^24g^ can be observed for samples with excess Cu. On the other hand, for the Cu^12e^ position (Figure 10b), the lengths of the bonds with directly neighboring S^2a^ and S^24g^ atoms decrease or remain practically unchanged, while the distances to the further surrounding Sb and Cu^12e^ atoms increase, which is the result of an increase in the unit cell size. Compared to the sample containing Cu in the 24g void [16], a smaller increase in bond length with S^24g^ and a larger increase in distance to Cu^12e^ can be observed. If we look at the void □^6b^ (Figure 10c), we observe that the distances to all atoms in the nearest neighborhood, i.e., four S^24g^, four Cu^12d^, two Cu^12e^ atoms, increase to a much larger extent for HLC phases than for LLC phases. The uniform increase in distance is consistent with the high symmetry of this void. A similar effect was observed in electronic structure calculations for structures containing Mg^6b^ (Figure 4a). On the other hand, it can be seen that for structures with excessive Cu calculated by the DFT method (Figure 4c), the increase in distance between the □^6b^ and S^24g^ is significantly smaller. For the void □^24g^ (Figure 10d), the changes in distance from the nearest atoms are less uniform. The shift relative to the closer two S^24g^ atoms is twice as small as for the third further S^24g^ atom, which is unlikely to be the case if this void is occupied by an Mg atom or Cu atom. If we compare the observed shifts of bond lengths for particular positions (Figure 10a–d) with those for coordination polyhedra, which result from DFT calculations (Figure 4a–d) where the largest atomic shifts for Mg in 24g site were predicted for Sb and Cu^12e^ atoms, we can reject the case of Cu/Mg in □^24g^. A similar comparison of atomic shifts for the substitution of Cu^12e^ and Cu^12d^ atoms with Mg, which for the DFT calculated structures lead to decreases in distance around Mg atoms, seems not to be very likely.

Comparison of the atomic shifts observed in the XRD results with the atomic shift results obtained for the structural parameters obtained in the DFT calculations indicates that in the case of the HLC phase in Mg_x_Cu_12_Sb_4_S_13_, the observations most closely match the model in which Mg occupies □^6b^.

### 3.3. SEM and EDS Analysis

**Figure 11:**
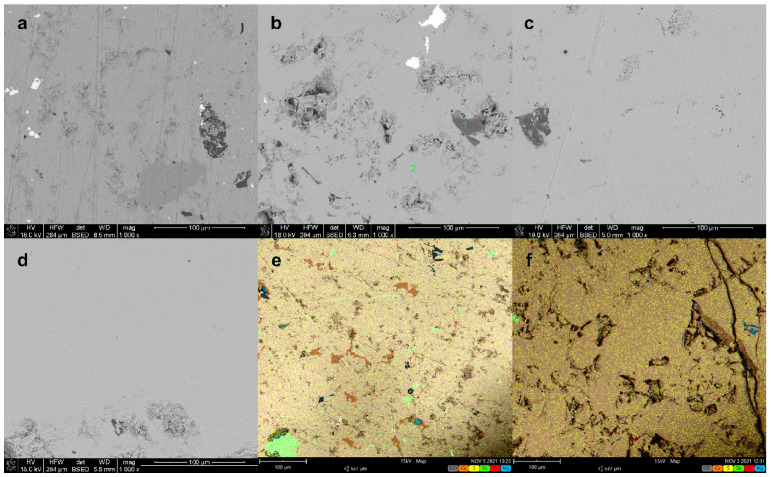
Scanning electron micrographs of Mg_x_Cu_12_Sb_4_S_13_ sintered samples. (**a**) Mg_0_._5_Cu_12_Sb_4_S_13_; (**b**) Mg_1_._0_Cu_12_Sb_4_S_13_; (**c**) Mg_1_._5_Cu_12_Sb_4_S_13_; (**d**) Mg_2_._0_Cu_12_Sb_4_S_13_; (**e**,**f**) Mg_0_._5_Cu_12_Sb_4_S_13_, Mg_1_._0_Cu_12_Sb_4_S_13_ back-scattered electrons SEM image combined with EDS analysis.

SEM observations show that the samples obtained are dense and consist mostly of the phase corresponding to tetrahedrite stoichiometry, but have precipitates of various impurities (Figure 11). These impurities are mainly Cu_2_S and MgS in the case of Mg_0_._5_Cu_12_Sb_4_S_13_ sample, and Sb, MgS and MgO in the cases of the Mg_1_._0_Cu_12_Sb_4_S_13_ and Mg_1_._5_Cu_12_Sb_4_S_13_ samples. The appearance of foreign phase precipitates is often observed during the doping of tetrahedrites [11]. The most common are Cu_3_SbS_4_ and CuSbS_2_ and compounds formed by the dopant. A surprising dependence of the amount of impurities on the amount of dopant was observed in the case of previous work concerning Mg doping, where the amount of precipitates, which are Cu_3_SbS_4_, CuSbS_2_ and MgS, decreases with the increase in the Mg amount [7]. Surprisingly, as in study [7], the amount of precipitates observed in our samples decreases with increasing Mg content. In the case of the sample Mg_2_._0_Cu_12_Sb_4_S_13_, it was almost completely devoid of visible impurities, while cracks at the grain boundaries were clearly visible. In the case of this sample, it was observed that after a few days, numerous cracks formed in the sample and after a few weeks, complete disintegration into tiny pieces occurred. Therefore, in the further analysis of chemical composition and microstructure, this sample was omitted. EDS chemical composition analysis showed that Mg was present in a phase with a chemical composition corresponding to tetrahedrite in the vast majority of point analyses, confirming the incorporation of magnesium into the tetrahedrite structure. The contents of the elements were normalised with respect to sulphur. The results obtained are presented in Table 2. In selected cases, where precipitates of MgS and Cu_2_S were visible in the analysed area, they were used as an internal standard to correct the calculated stoichiometry of the composition, except for the Sb content.

The Cu:S ratio after correction relative to the standard was close to the 12:13 ratio for pure tetrahedrite. The observed Mg content, on the other hand, was almost twice as high as the assumed Mg content, so the quantitative result of this analysis should be treated with caution and assumed to be overestimated despite the correction. As can be seen from the results presented in Table 2, in the case of measurements which were not corrected with respect to the internal standard, the copper content was definitely overestimated with respect to the corrected results. For the remaining samples, the copper content exceeded the nominal content. In addition, the spread of the observed Cu contents is greater than the mean absolute deviation. In our opinion, this scatter is more a result of systematic errors occurring during the EDS analysis than of large variations in the chemical composition of the samples. In the case of magnesium content, almost all results are within the measurement error and the content is about x = 1. It can be seen that the actual Mg content decreases slightly with increasing nominal Mg content, which corresponds to the observed decrease in lattice parameter and increase in the content of the LLC phase. The most likely explanation for this phenomenon is the loss of magnesium from the synthesised material due to the reaction between the magnesium and the quartz ampoule observed during the first stage of synthesis. In the case of samples with higher magnesium content, the results of this process were observed to a greater extent, which may also be responsible for the reduction in the quantity of impurity phases. The point analysis of the composition did not allow the distinction of HLC and LLC phases on the basis of the composition analysis. No clear correlation between Mg and Cu contents was observed, which could indicate that Mg substitutes Cu, but this possibility cannot be excluded either. The corrected Mg and Cu content results for sample Mg_0_._5_Cu_12_Sb_4_S_13_ suggest that magnesium most probably occupies structural voids, which is in agreement with the results of DFT calculations. The very similar Mg content for all samples is also in agreement with the DFT calculation results, which indicate the existence of an energetically optimal Mg content in the 6b void. Unfortunately, differences in the results of quantitative analysis of composition obtained in point analysis of different areas are much larger than the fluctuations of composition in different grains visible in one analysed area. This does not allow us to unambiguously determine the stoichometry of the tetrahedrite phase in the obtained samples and, at the same time, the localization of Mg.

### 3.4. Electrical Properties

**Figure 12:**
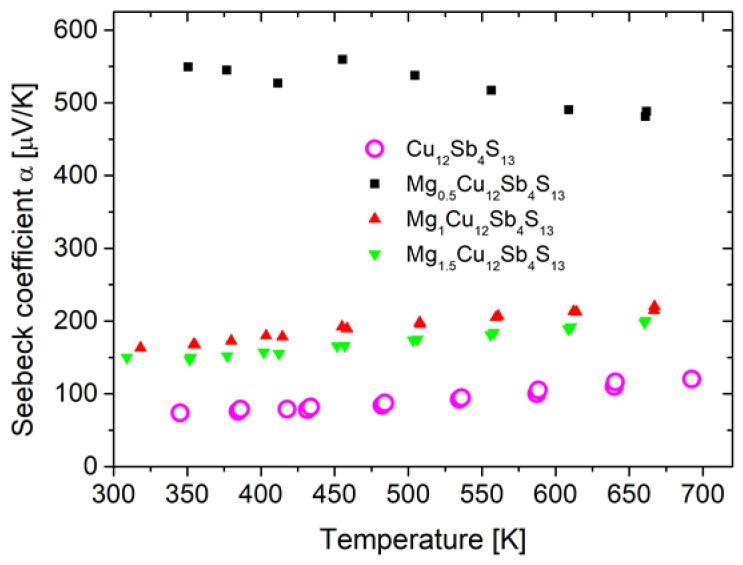
Temperature dependence of the Seebeck coefficient for Mg_x_Cu_12_Sb_4_S_13_ sintered samples.

Electrical measurements were performed for a reference sample of pure tetrahedrite, and for samples with nominal magnesium contents x = 0.5, 1.0, 1.5. The sample with Mg content x = 2.0, due to its decomposition, was not subjected to electrical property measurements. The measured values of the Seebeck coefficient of Mg-doped samples and their temperature dependence are presented in Figure 12. The Seebeck coefficient for all of the measured specimens has positive values throughout the whole measurement range, which indicates the *p*-type nature of the materials obtained. For pure tetrahedrite, the Seebeck coefficient value at room temperature is close to 80 μV/K, which is very close to the values reported by Chetty et al. [4], Levinsky et al. [7], and Vaqueiro et al. [16], and slightly lower than in the works of Kumar et al. [3] and Barbier et al. [5]. Mg doping changes the dependence of the Seebeck coefficient for the Mg_0_._5_Cu_12_Sb_4_S_13_ sample, which becomes inversely proportional to temperature and, at the same time, the values of the Seebeck coefficient increase several times compared to the pure tetrahedrite sample. For samples with higher Mg content, which show presence of phase with low lattice parameter, the Seebeck coefficient decreases with increase in the Mg content, showing temperature dependence similar to pure tetrahedrite. An increase in Seebeck coefficient during Mg doping was also observed by Levinski et al. [7]; however, the maximum values that were reported are much lower. Since most of the dopants used in optimizing the thermoelectric properties of tetrahedrites are aimed at reducing the carrier concentration, i.e., hole compensation, an increase in the Seebeck coefficient during doping is observed for most of the dopants studied. In most of the works found in the literature, the maximum Seebeck coefficient values reach around 200 μV/K. The highest values reported were observed for Co [14] and Mn [11] doping and were 300 and 400 μV/K, respectively. For most of the materials studied, the Seebeck coefficient at temperatures above room temperature increases almost linearly with temperature. Only in the work of Vaqueiro et al. [16], in which excess copper was introduced into the structural voids, a more complex dependence was observed, where up to a certain temperature (phase transition temperature), the value of the Seebeck coefficient decreases to undertake a dependence typical of tetrahedrites above this temperature.

**Figure 13:**
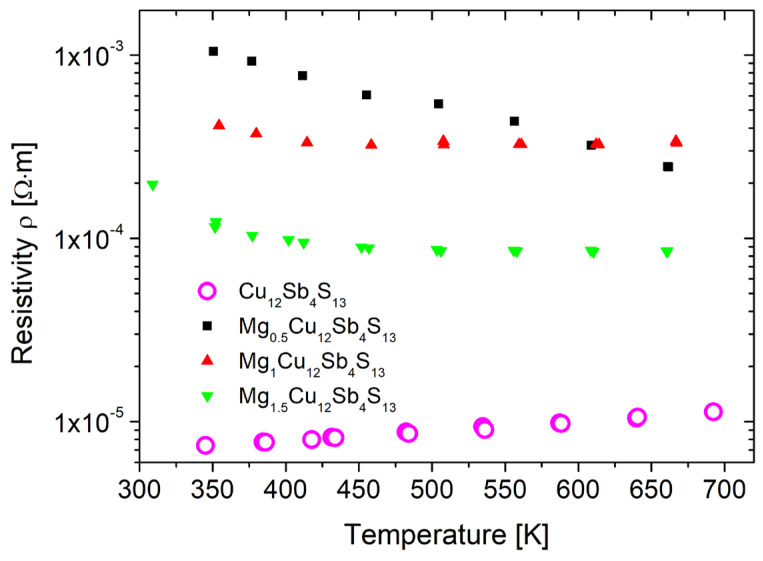
Temperature dependence of electrical resistivity for Mg_x_Cu_12_Sb_4_S_13_ sintered samples.

The results of electrical resistivity measurements are shown in Figure 13. The electrical resistivity of pure tetrahedrite has a metallic-like temperature dependence and decreases slightly with temperature. The resistance values are similar to those reported in the literature. The doping with Mg causes a pronounced increase in resistance, the maximum of which is observed for the Mg_0_._5_Cu_12_Sb_4_S_13_ sample. At the same time, the character of the temperature dependence changes to semiconductor-like. Such dependence may result from the compensation of holes by electrons provided by Mg atoms built into the tetrahedrite structure. As can be supposed from the results presented above, the HLC tetrahedrite phase has Mg built into the voids 6b, which according to the electronic structure calculations provides two electrons per formula unit, in contrast to the phase where Mg is replacing mono- or divalent Cu, and can provide a maximum of 1 e/f.u. This hypothesis is also consistent with the results of the Seebeck coefficient where high value of the Seebeck coefficient for Mg_0_._5_Cu_12_Sb_13_ is probably a result of the strong compensation of holes by electrons donated by Mg and consistent with the results of the DFT calculations. The addition of a larger than x = 0.5 amount of magnesium causes a decrease in resistivity, but it is still higher than for pure tetrahedrite. Such a result can be linked to a change in the ratio of the two tetrahedrite phases and increasing content of the tetrahedrite phase with the smaller lattice constant, which is likely to contain no or less Mg filling the structural voids, resulting in a higher hole concentration and lower resistivity. The nature of the observed changes in electrical properties indicates that Mg behaves as a typical donor dopant, especially in the case of a HLC phase, which is consistent with theoretical predictions for the filling of the 6b structural voids by Mg atoms.

## 4. Conclusions

We obtained a series of tetrahedrites Mg_x_Cu_12_Sb_4_S_13_ with excess Mg, which, as shown by structural analysis, at room temperature, consist mostly of one or two phases with a tetrahedrite structure. The first of these phases, HLC, has a lattice constant of about 10.45 Å and the second, LLC, whose weight content increases with the amount of Mg, has a lower lattice constant of about 10.37 Å. These tetrahedrite phases are accompanied by impurities of Sb, MgS, Cu_2_S and MgO.

DFT ab initio calculations for tetrahedrite structures containing excess copper in the 6b and 24g voids, Cu_12+x_Sb_4_S_13_, showed that by far the energetically preferred location of Cu is the structural void at the 24g position. Similarly performed calculations for the tetrahedrite structures Mg_x_Cu_12_Sb_4_S_13_ with excess Mg, supported by the experimental results, show that the most probable location of Mg is the void in the Wyckoff’s position 6b (0, 0, ½).

The experimental results do not unambiguously exclude other locations of the dopant, so it is possible that a partial substitution of Cu by Mg and occupation of some Cu in voids at the 24g position occur simultaneously. Presumably, the two phases of tetrahedrite, which are observed in some of the samples, have different types of defects, as can be seen from the slightly different directions of the atomic shifts. The insertion of Mg atoms into the structural void 6b is, as suggested by the experimental results, most likely for the HLC phase with a higher lattice constant.

The theoretical calculations of the formation energy of Mg_x_^6b^Cu_12_Sb_4_S_13_ showed that there should be a limit of solubility of Mg in the tetrahedrite structure at x = 0.5~1. These predictions are mainly confirmed by the results of EDS analysis, which showed that the observed Mg content in the samples is more or less constant.

The electrical results show that Mg behaves as a donor dopant. The high value of the Seebeck coefficient indicates that by introducing Mg into the tetrahedrite structure (presumably into the voids), it was possible to significantly reduce the concentration of holes. Unfortunately, due to the existence of a solubility limit of Mg of below x = 1, tetrahedrites of *n*-type conductivity could not be obtained. However, finding another filler atom with a higher filling fraction limit or combining magnesium with another donor dopant can lead to *n*-type tetrahedrites.

## Figures and Tables

**Table 1:** Global instability index *D*, global bond strain index δ* and bond strain index *δ* calculated for Mg_x_^6b^Cu_12_Sb_4_S_13_ structures.

	x = 0.5	x = 1	x = 1.5	x = 2	x = 3
*D*	0.110	0.155	0.173	0.193	0.199
*δ* *	0.059	0.079	0.114	0.135	0.192
*δ* (Cu_1_–Sb)	0.088	0.119	0.191	0.240	0.356
*δ* (S_3_–Cu_1_)	0.035	0.032	0.055	0.085	0.139
*δ* (S_1_–Cu_3_)	0.068	0.092	0.094	0.092	0.088
*δ* (S_1_–Sb)	0.031	0.044	0.081	0.130	0.201
*δ* (S_1_–Cu_1_)	0.027	0.038	0.051	0.071	0.101

**Table 2:** Stoichiometric coefficients of Mg_x_Cu_y_Sb_z_S_13_ tetrahedrite calculated from results of EDS point analysis.

	Mg	*d*_aver_	Cu	*d*_aver_	Sb	*d*_aver_	S
Mg_0.5_Cu_12_Sb_4_S_13_ * I ^i^	0.89	0.1	11.9	0.3	5.55	0.06	13
Mg_0.5_Cu_12_Sb_4_S_13_ * II ^i^	0.87	0.1	11.8	0.5	5.52	0.07	13
Mg_0.5_Cu_12_Sb_4_S_13_ I ^i^	1.1	0.1	13.9	0.3	5.55	0.06	13
Mg_0.5_Cu_12_Sb_4_S_13_ II ^i^	1.06	0.14	13.7	0.5	5.52	0.07	13
Mg_0.5_Cu_12_Sb_4_S_13_ III ^i^	1.0	0.15	13.9	0.4	5.57	0.05	13
Mg_1.0_Cu_12_Sb_4_S_13_ I ^i^	0.79	0.15	12.0	0.3	5.29	0.09	13
Mg_1.0_Cu_12_Sb_4_S_13_ II ^ii^	1.0	0.26	12.2	0.3	5.16	0.12	13
Mg_1.5_ Cu_12_Sb_4_S_13_ I ^ii^	0.76	0.14	13.9	0.3	5.42	0.07	13
Mg_1.5_Cu_12_Sb_4_S_13_ II ^i^	0.65	0.1	12.8	0.3	5.33	0.08	13

* Results where Cu:S ratio and Mg:S ratio were corrected using Cu_2_S and MgS precipitates as internal reference; ^i^—results averaged for 12 points; ^ii^—results averaged for 24 points; *d*_aver_—mean absolute deviation.

## Data Availability

Not applicable.

## Supplementary Materials

The following supporting information can be downloaded at: https://www.mdpi.com/article/10.3390/ma15124115/s1, Figure S1: Band structure of MgxCu12Sb4S13 for different content of Mg in the 6b void. For clarity only four bands closest to the band gap are shown. Dashed line shows Fermi energy level. Figure S2: Plots of the relative change in bond length or distance ∆l upon doping with Mg measured at different atomic sites for Mg_1_Cu_12_Sb_4_S_13_. Results are expressed as a percentage of a given bond/distance. * Results for Cu_12.3_Sb_4_S_13_ from [16].

## References

[1] Weller D., Morelli D.T. (2017). Rapid synthesis of zinc and nickel co-doped tetrahedrite thermoelectrics by reactive spark plasma sintering and mechanical alloying. J. Alloy. Compd..

[2] Suekuni K., Tsuruta K., Kunii M., Nishiate H., Nishibori E., Maki S., Ohta M., Yamamoto A., Koyano M. (2013). High-performance thermoelectric mineral Cu_12−x_Ni_x_Sb_4_S_13_ tetrahedrite. J. Appl. Phys..

[3] Kumar D.S., Chetty R., Rogl P., Rogl G., Bauer E., Malar P., Mallik R.C. (2016). Thermoelectric properties of Cd doped tetrahedrite: Cu_12−x_Cd_x_Sb_4_S_13_. Intermetallics.

[4] Chetty R., Bali A., Naik M.H., Rogl G., Rogl P., Jain M., Suwas S., Mallik R.C. (2015). Thermoelectric properties of Co substituted synthetic tetrahedrite. Acta Mater..

[5] Barbier T., Lemoine P., Gascoin S., Lebedev O.I., Kaltzoglou A., Vaqueiro P., Powell A.V., Smith R.I., Guilmeau E. (2015). Structural stability of the synthetic thermoelectric ternary and nickel-substituted tetrahedrite phases. J. Alloy. Compd..

[6] Sun F.H., Wu C.F., Li Z., Pan Y., Asfandiyar, Dong J., Li J.F. (2017). Powder metallurgically synthesized Cu_12_Sb_4_S_13_ tetrahedrites: Phase transition and high thermoelectricity. RSC Adv..

[7] Levinsky P., Candolfi C., Dauscher A., Lenoir B., Hejtmanek J. (2019). Thermoelectric Properties of Magnesium-Doped Tetrahedrite Cu_12−x_Mg_x_Sb_4_S_13_. J. Electron. Mater..

[8] Lu X., Morelli D.T., Xia Y., Zhou F., Ozolins V., Chi H., Zhou X., Uher C. (2013). High Performance Thermoelectricity in Earth-Abundant Compounds Based on Natural Mineral Tetrahedrites. Adv. Energy Mater..

[9] Heo J., Laurita G., Muir S., Subramanian M.A., Keszler D.A. (2014). Enhanced Thermoelectric Performance of Synthetic Tetrahedrites. Chem. Mater..

[10] Bouyrie Y., Candolfi C., Pailhès S., Koza M.M., Malaman B., Dauscher A., Tobola J., Boisron O., Saviot L., Lenoir B. (2015). From crystal to glass-like thermal conductivity in crystalline minerals. Phys. Chem. Chem. Phys..

[11] Chetty R., Kumar P., Rogl G., Rogl P., Bauer E., Michor H., Suwas S., Puchegger S., Giesterg G., Mallik R.C. (2015). Thermoelectric properties of a Mn substituted synthetic tetrahedrite. Phys. Chem. Chem. Phys..

[12] Bouyrie Y., Candolfi C., Ohorodniichuk V., Malaman B., Dauscher A., Tobola J., Lenoir B. (2015). Crystal structure, electronic band structure and high-temperature thermoelectric properties of Te-substituted tetrahedrites Cu_12_Sb_4−x_Te_x_S_13_ (0.5 ≤ x ≤ 2.0). J. Mater. Chem. C.

[13] Kumar D.S.P., Chetty R., Femi O.E., Chattopadhyay K., Malar P., Mallik R.C. (2017). Thermoelectric Properties of Bi Doped Tetrahedrite. J. Electron. Mater..

[14] Lu X., Morelli D. (2014). The Effect of Te Substitution for Sb on Thermoelectric Properties of Tetrahedrite. J. Electron. Mater..

[15] Lu X., Morelli D.T., Wang Y., Lai W., Xia Y., Ozolins V. (2016). Phase Stability, Crystal Structure, and Thermoelectric Properties of Cu_12_Sb_4_S_13–x_Se_x_ Solid Solutions. Chem. Mater..

[16] Vaqueiro P., Guelou G., Kaltzoglou A., Smith R.I., Barbier T., Guilmeau E., Powell A.V. (2017). The Influence of Mobile Copper Ions on the Glass-Like Thermal Conductivity of Copper-Rich Tetrahedrites. Chem. Mater..

[17] Suekuni K., Tsuruta K., Ariga T., Koyano M. (2012). Thermoelectric Properties of Mineral Tetrahedrites Cu_10_Tr_2_Sb_4_S_13_ with Low Thermal Conductivity. Appl. Phys. Express..

[18] Lu X., Morelli D.T. (2013). Rapid synthesis of high-performance thermoelectric materials directly from natural mineral tetrahedrite. MRS Commun..

[19] Kosaka Y., Suekuni K., Hashikuni K., Bouyrie Y., Ohta M., Takabatake T. (2017). Effects of Ge and Sn substitution on the metal–semiconductor transition and thermoelectric properties of Cu_12_Sb_4_S_13_ tetrahedrite. Phys. Chem. Chem. Phys..

[20] Makovicky E., Skinner B.J. (1979). Studies of the sulfosalts of copper; VI, Low-temperature exsolution in synthetic tetrahedrite solid solution, Cu_12+x_Sb_4+y_S_13_. Can. Mineral..

