# Supplementary material for: Experimental and Theoretical Studies on Possibility of Void Filling by Magnesium in Mg-Doped Tetrahedrites
Source: Materials (Basel). 2022 Jun 9;15(12):4115. doi: 10.3390/ma15124115 (PMC9228319; doi:10.3390/ma15124115)
Supplement: Supplementary file 1 [file materials-15-04115-s001.zip › materials-1740850-supplementary.pdf]

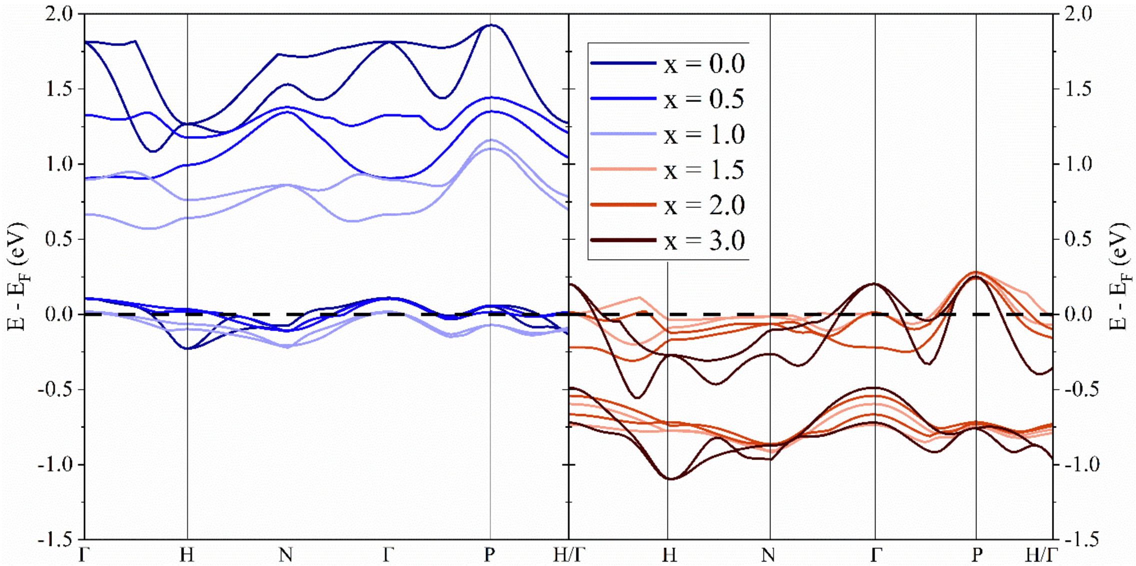

Figure S1. Band structure of $Mg_xCu_{12}Sb_4S_{13}$ for different content of Mg in the 6b void. For clarity only four bands closest to the band gap are shown. Dashed line shows Fermi energy level.

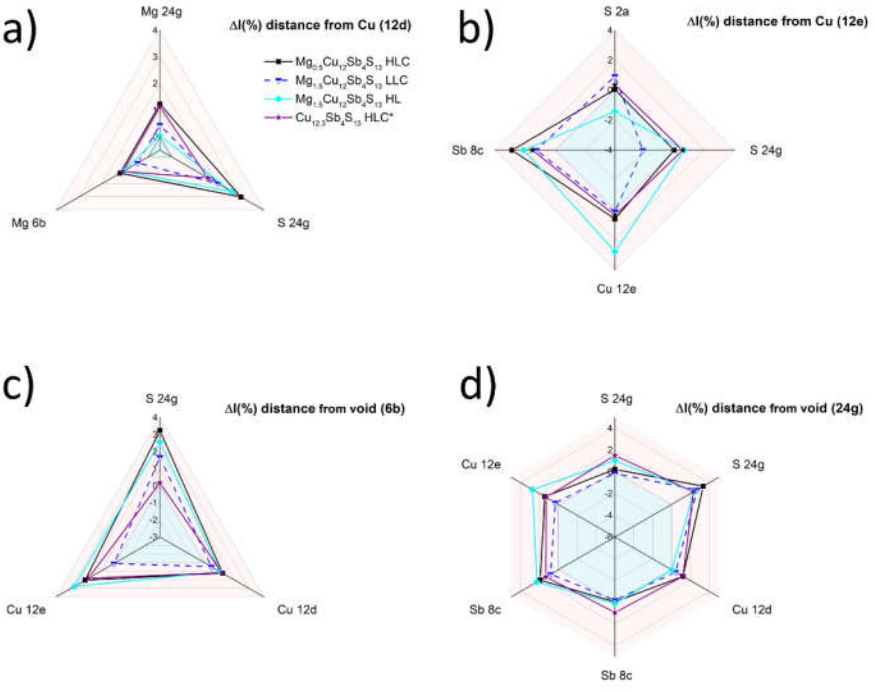

Figure S2 Plots of the relative change in bond length or distance Δl upon doping with Mg measured at different atomic sites for $Mg_1Cu_{12}Sb_4S_{13}$. Results are expressed as a percentage of a given bond/distance. *Results for $Cu_{12.3}Sb_4S_{13}$ from [16].
